# Extracellular histones disarrange vasoactive mediators release through a COX‐NOS interaction in human endothelial cells

**DOI:** 10.1111/jcmm.13088

**Published:** 2017-02-28

**Authors:** Daniel Pérez‐Cremades, Carlos Bueno‐Betí, José Luis García‐Giménez, José Santiago Ibañez‐Cabellos, Carlos Hermenegildo, Federico V. Pallardó, Susana Novella

**Affiliations:** ^1^ Department of Physiology Faculty of Medicine and Dentistry University of Valencia Valencia Spain; ^2^ INCLIVA Biomedical Research Institute Valencia Spain; ^3^ Center for Biomedical Network Research on Rare Diseases (CIBERER) Institute of Health Carlos III Valencia Spain

**Keywords:** extracellular histones, endothelial cells, vascular mediators, nitric oxide, prostanoids

## Abstract

Extracellular histones are mediators of inflammation, tissue injury and organ dysfunction. Interactions between circulating histones and vascular endothelial cells are key events in histone‐mediated pathologies. Our aim was to investigate the implication of extracellular histones in the production of the major vasoactive compounds released by human endothelial cells (HUVECs), prostanoids and nitric oxide (NO). HUVEC exposed to increasing concentrations of histones (0.001 to 100 μg/ml) for 4 hrs induced prostacyclin (PGI2) production in a dose‐dependent manner and decreased thromboxane A2 (TXA2) release at 100 μg/ml. Extracellular histones raised cyclooxygenase‐2 (COX‐2) and prostacyclin synthase (PGIS) mRNA and protein expression, decreased COX‐1 mRNA levels and did not change thromboxane A2 synthase (TXAS) expression. Moreover, extracellular histones decreased both, eNOS expression and NO production in HUVEC. The impaired NO production was related to COX‐2 activity and superoxide production since was reversed after celecoxib (10 μmol/l) and tempol (100 μmol/l) treatments, respectively. In conclusion, our findings suggest that extracellular histones stimulate the release of endothelial‐dependent mediators through an up‐regulation in COX‐2‐PGIS‐PGI2 pathway which involves a COX‐2‐dependent superoxide production that decreases the activity of eNOS and the NO production. These effects may contribute to the endothelial cell dysfunction observed in histone‐mediated pathologies.

## Introduction

Recent studies indicate that histones mediate proinflammatory activity when are released into extracellular space 1. In this regard, high levels of circulating histones in plasma have been detected in trauma‐associated injury 2, ischaemia–reperfusion injuries in kidney 3 liver 4 and sepsis 5, 6.

Endothelium participates in numerous regulatory functions and contributes to and is affected by inflammatory processes. It is also involved in blood coagulation and fibrinolysis, immune response by modulation of leucocyte interactions with the vessel wall and regulation of vascular tone and blood pressure 7. Disturbance of the endothelium functional integrity in response to circulating compounds reflects a first step in many disorders. After pro‐inflammatory stimuli, endothelium undergoes activation characterized by increased local blood flow, leakage of plasma‐protein‐rich fluid into the tissues and recruitment and activation of circulating leucocytes 8.

Endothelium exerts these actions through the release of vasoactive compounds, including prostanoids and nitric oxide (NO), that control functions of both vascular smooth muscle cells and of circulating blood cells 7.

Cyclooxygenases (COX) are the rate‐limiting enzymes in the production. COX isoenzyme (COX‐1 and COX‐2) expression has been detected in the vascular system 9. COX‐1 is considered the constitutive isoform in endothelium, while COX‐2 is induced under pro‐inflammatory conditions. However, both COX share characteristics of constitutive and inducible enzymes in endothelial cells 10. On the other hand, NO is mainly produced by endothelial nitric oxide synthase (eNOS) in the vasculature 11. NO dilates blood vessels through acting on smooth muscle cells and inhibits platelet aggregation. NO also decreases leucocyte adhesion by suppressing cell adhesion molecule expression on cell membrane surface, therefore contributing to quiescence of resting endothelial cells 12.

Endothelial cells exposed to extracellular histones release pro‐inflammatory cytokines, induce tissue factor expression 13 and increase adhesion molecules in the cell membrane 14. However, the vasoactive effect of extracellular histones has been less studied. Therefore, the aim of this study was to describe the implication of extracellular histones in the release of the two major vasoactive compounds released by endothelial cells, NO and prostanoids.

## Materials and methods

### Cell culture and experimental design

Primary human umbilical vein endothelial cell (HUVEC) cultures were obtained from human umbilical cord from ‘Hospital Clínico Universitario’ of Valencia as previously described 15. Briefly, umbilical veins were treated with 1% collagenase (Life Technologies, Carlsbad, CA, USA), and HUVECs extracted were cultured in specific endothelial growth medium, EGM‐2 (Lonza, Cultek, Barcelona, Spain).

HUVECs from passages 3 to 5 were used in this study. When they reached confluence, media were changed and cells were exposed during 4 hrs to different calf thymus (CT) histone concentrations (Sigma‐Aldrich, St. Louis, MO, USA): 1, 10, 100 ng/ml and 1, 10, 25, 50 and 100 μg/ml prepared in PBS and free of LPS. In some experiments, 10 μmol/l celecoxib, a specific COX‐2 inhibitor (Sigma‐Aldrich) and 100 μmol/l tempol (Sigma‐Aldrich), a superoxide dismutase mimetic, were added to HUVEC 1 hr before histone treatments.

Cells were identified as endothelial by their characteristic cobblestone morphology and the presence of von Willebrand factor by immunofluorescence using a specific antibody (ab6994; Abcam, Cambridge, UK). Cells used in this study were more than 95% vWF positive.

Cell viability was measured by flow cytometry using propidium iodide (Immunostep, Salamanca, Spain). Our results showed that extracellular histones scarcely affect HUVEC viability. Only at higher concentrations, extracellular histones induce a mortality of 6.4% (50 μg/ml) and 10.9% (100 μg/ml) respect to non‐treated cells.

The investigation conforms to the principles outlined in the Declaration of Helsinki and was approved by the Ethical Committee of Clinical Research of the INCLIVA, ‘Hospital Clínico Universitario’ of Valencia, and written informed consent was obtained from all donors.

### Nitric oxide measurement

NO production was determined by fluorescence microscopy and 4‐amino‐5‐methylamino‐2′,7′‐difluorofluorescein diacetate (DAF‐FM Diacetate) (Life Technologies, Alcobendas, Madrid, Spain) probe. After 3‐hrs treatment, DAF‐FM was added to culture media and incubated for 45 min. Media were then replaced, and cells were incubated for 15 additional minutes to ensure complete fluorescence probe deacetylation.

NO production was determined by measuring intensity fluorescence at 515 nm on an inverted fluorescence microscope (Eclipse Ti‐S; Nikon Co, Tokyo, Japan). Three randomly selected pictures per condition tested were taken, and fluorescence intensity measurements were recorded using NIS‐Elements 3.2 software (Nikon Co). Results are presented as the mean intensity fluorescence per power field subtracting the background and were relativized to non‐treated cells.

### Prostacyclin and thromboxane A2 determination

The amount of PGI2 and TXA2 produced was measured by enzyme immune assay using commercial EIA kits (Cayman Chemical, Ann Arbor, MI, USA) as previously described 16. After treatments, media were collected and stored at −80°C. Cells were lysed in RIPA buffer (Sigma‐Aldrich) for protein determination, calculated by the Pierce BCA protein assay kit using BSA as a standard (Thermo Scientific Inc., Rockford, USA).

PGI2 and TXA2 levels were calculated as the concentration of stable hydrolysis metabolite products, 6‐keto‐prostaglandin‐F1alpha and TXB2, respectively. Results were expressed as the ratio increases over untreated control in ng prostanoid/mg protein.

### RNA isolation and quantitative real‐time PCR assay (qRT‐PCR)

Total RNA was extracted using TRIzol reagent (Invitrogen, Barcelona, Spain) following the manufacturer's instructions. Reverse transcription of 200 ng of total RNA was carried out using High‐Capacity cDNA Reverse Transcription Kit (Applied Biosystems, Foster City, CA, USA) using Mastercycler Eppendorf Thermocycler (Eppendorf, Hamburg, Germany). The mRNA levels were determined by qRT‐PCR analysis using an ABI Prism 7900 HT Fast Real‐Time PCR System (Applied Biosystems). Gene‐specific primer pairs and probes were purchased from Thermo Fisher (Assay‐on‐Demand) for eNOS (Hs01574659_m1), COX‐1 (Hs00377726_m1), COX‐2 (Hs00153133_m1), PGIS (Hs00919949_m1) TXAS (Hs00233423_m1) and GAPDH (endogenous control, Hs99999905_m1) and were used with TaqMan Universal Mastermix (Thermo Fisher, Rockford, IL, USA). PCR conditions were 10 min. at 95°C for enzyme activation, followed by 40 two‐step cycles (15 sec. at 95°C; 1 min. at 60°C). Data were analysed with the SDS 2.2.2 software (Applied Biosystems) according to the 2^−ΔΔ*C*t^ method.

### Western blot

Treated HUVECs were collected in RIPA buffer (Sigma‐Aldrich) containing protease and phosphatase inhibitors (Roche Diagnostics, Madrid, Spain). Protein content was measured by the Pierce BCA protein assay kit (Thermo Scientific Inc.) using BSA as a standard. Equal amounts of protein were then separated by SDS‐polyacrylamide gel electrophoresis and transferred to nitrocellulose membranes (Whatman, GE Healthcare Life Sciences, Chicago, IL, USA). Immunostaining was achieved using specific antibodies: eNOS (sc‐653), COX‐1 (sc‐19998), COX‐2 (sc‐19999), PGIS (sc‐20933), TXAS (sc‐79181) all from Santa Cruz BioTechnology (Heidelberg, Germany) and β‐actin (Sigma‐Aldrich) as loading control. Development was performed peroxidase‐linked secondary antibodies (Santa Cruz Biotechnology). Luminol (ECL Western Blotting Detection Reagents, GE Healthcare, Hatfield, and Hertfordshire, UK) was added onto the membrane, and membranes were revealed by an image reader LAS‐4000 (GE Healthcare, Uppsala, Sweden). Signal density was analysed with ImageJ software (NIH Image, National Institutes of Health, Bethesda, MD, USA).

### Superoxide determination

Intracellular superoxide concentration was detected by measuring dihydroethidium (DHE) oxidation. DHE enters in the cell and is oxidized by superoxide to yield ethidium. Binding of ethidium to DNA produces red fluorescence. Histone‐treated cells were loaded with 2.5 μmol/l DHE for 30 min. Then, cells were rinsed with PBS and observed under an inverted fluorescence Nikon Eclipse Ti‐S microscope. Fluorescence from three different fields per well was measured (excitation wavelength: 490 nm; emission wavelength: 610 nm). Fluorescence signals were quantified using NIS‐Elements 3.2 software (Nikon Izasa S.A, L'Hospitalet de Llobregat, Spain).

### Statistical analysis

Values are expressed as mean ± S.E.M. A one‐way analysis of variance was used to determine the difference between groups. When an interaction effect was found, multiple comparisons were made using the Tukey method, and ‘*post hoc*’ test was performed. The significance has been considered at *P < 0.05, **P < 0.01 and ***P < 0.001, as indicated in each case. GraphPad Prism v5.0 (GraphPad Software, San Diego, CA, USA) was used for statistical analysis and graphic representations.

## Results

### Effect of extracellular histones on the endothelial production of NO, PGI2 and TXA2

The first objective of this work was to investigate the effect of extracellular histones on the endothelial production of vasoactive compounds, in particular the two main vascular prostanoids, PGI2 and TXA2, and NO. HUVECs were exposed for 4 hrs to increasing concentration of extracellular histones (1, 10 and 100 ng/ml and 1, 10, 25, 50 and 100 μg/ml).

**Figure 1:**
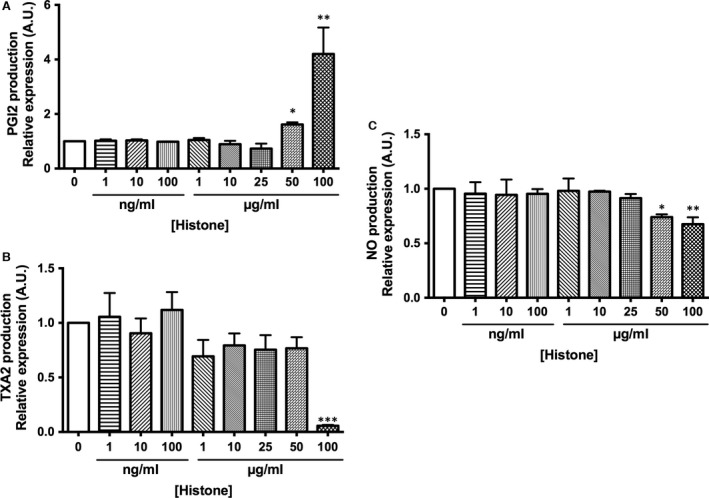
Extracellular histone‐treated HUVEC alter PGI2 and TXA2 release and decrease NO production. (**A** and **B**) HUVECs were exposed to different concentrations of histones for 4 hrs. Cultured medium was then collected, and PGI2 and TXA2 concentration was measured by enzyme immunoassay. Data are expressed as mean ± S.E.M. of *n* = 8–10 from three to five independent experiments. (**C**) HUVEC incubated with different concentrations of histones for 4 hrs were preloaded for 40 min. with the NO probe DAF‐FM for NO production determination. Data are expressed as mean ± S.E.M. of *n* = 6–8 from three to five independent experiments. *P < 0.05; **P < 0.01; ***P < 0.001 *versus* histones 0 μg/ml.

No effect was observed in PGI2 production at low concentrations of histones from 1 ng/ml to 25 μg/ml. However, the production of PGI2 increased in a dose‐dependent manner at 50 and 100 μg/ml (*P* < 0.05). This increment was up to 62 ± 8% in cells exposed to 50 μg/ml and up to 420 ± 97% in cells exposed to 100 μg/ml, compared to non‐treated cells (Fig. 1A). In contrast, the production of TXA2 by histone‐treated HUVEC decreased only at 100 μg/ml (*P* < 0.001) without changing at any other concentration assayed (Fig. 1B).

Using the same conditions described above, increasing concentrations of extracellular histones resulted in a significant decrease of NO production only at 50 (22 ± 3%, *P* < 0.05) and 100 μg/ml (26 ± 2%, *P* < 0.01) histones without changes after treatment from 1 ng/ml to 25 μg/ml (Fig. 1C).

### Effect of extracellular histones on gene and protein expression of prostanoid pathway

**Figure 2:**
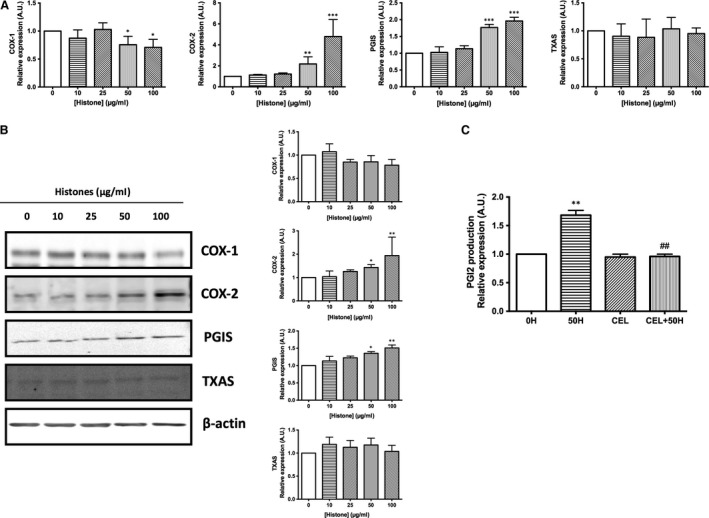
Extracellular histones alter HUVEC prostanoid production through up‐regulation of COX2‐PGI2 pathway. (**A**) HUVEC were exposed to 10–100 μg/ml of histones for 4 hrs. Relative COX‐1, COX‐2, PGIS, TXAS expression was determined by qRT‐PCR. Data are expressed as mean ± S.E.M. of *n* = 8–10 from three to five independent experiments. (**B**) Protein extracts (20 μg protein) from cultured HUVEC incubated at different concentrations of histones for 4 hrs were loaded on SDS‐PAGE gels and analysed by Western blotting using anti‐COX‐1, anti‐COX‐2, anti‐PGIS and anti‐TXAS. β‐actin was used as loading control. One representative experiment of three performed is shown. Relative levels assessed by densitometry are presented. (**C**) HUVECs were exposed to 50 μg/ml of histones (50H) for 4 hrs after pre‐incubation with celecoxib (CEL), a specific COX‐2 inhibitor. Cultured medium was then collected, and PGI2 concentration was measured by enzyme immunoassay. Data are expressed as mean ± S.E.M. of *n* = 3–4 from three independent experiments. *P < 0.05; **P < 0.01; ***P < 0.001 *versus* histones 0 μg/ml and ^*##*^ *P* < 0.01 *versus* histones 50 μg/ml.

mRNA and protein expression levels of the enzymes involved in PGI2 and TXA2 production were determined. Histone‐exposed HUVEC decreased COX‐1 mRNA in a dose‐dependent manner (Fig. 2A). At low concentrations of histones, from 10 to 25 μg/ml, mRNA COX‐1 did not change but the expression decreased up to 24 ± 7% when cells were exposed to 50 μg/ml (*P* < 0.05) and 29 ± 7% when cells were exposed to 100 μg/ml of histones (*P* < 0.05). However, COX‐2 mRNA expression increased up to 118 ± 19% at 50 μg/ml (*P* < 0.05) and 379 ± 66% at 100 μg/ml of histones (*P* < 0.001).

In the prostanoid pathway, the COX products cyclo‐endoperoxides PGG2 and PGH2 are rapidly converted in the active compounds PGI2 and TXA2 by means of the specific synthases PGIS and TXAS, respectively. Results from quantitative qRT‐PCR analysis showed differences in PGIS mRNA expression, while TXAS mRNA expression remained unaltered (Fig. 2A). Histone‐treated HUVEC increased PGIS mRNA levels up to 77 ± 5% at 50 μg/ml (*P* < 0.001) and up to 96 ± 6% at 100 μg/ml histones (*P* < 0.001). These data were in accordance with changes observed in PGI2.

Moreover, mRNA expression profile was supported with protein expression analysis determined by Western blot (Fig. 2B). HUVEC exposed to histones shown unaltered COX‐1 protein expression (although a tendency to decrease with higher histone concentrations exists), while COX‐2 was significantly increased when endothelial cells were exposed at 50 and 100 μg/ml histones. COX‐2 protein expression increased up to 43 ± 5% at 50 μg/ml histones (*P* < 0.05) and up to 94 ± 56% at 100 μg/ml histones (*P* < 0.01) above control values (Fig. 2B). Regarding specific synthases, mRNA PGIS levels were accompanied by an increment in the amount of PGIS protein expression, also at 50 μg/ml (36 ± 4%, *P* < 0.05) and 100 μg/ml histones (51 ± 6%, *P* < 0.01, Fig. 2B). Finally, no changes in TXAS protein expression were found (Fig. 2B).

Therefore, these results demonstrated that HUVECs exposed to extracellular histones show a modulation in COX pathway, mainly by an up‐regulation of COX‐2 and PGIS that, in turn, could cause an increase in PGI2 production. In fact, PGI2 production in 50 μg/ml histone‐treated endothelial cells in the presence of the selective COX‐2 inhibitor celecoxib (10 μmol/l) completely reversed the effect triggered by extracellular histones (Fig. 2C) supporting the involvement of COX‐2 in the observed effect.

### Effect of extracellular histones on gene and protein expression of NO pathway

**Figure 3:**
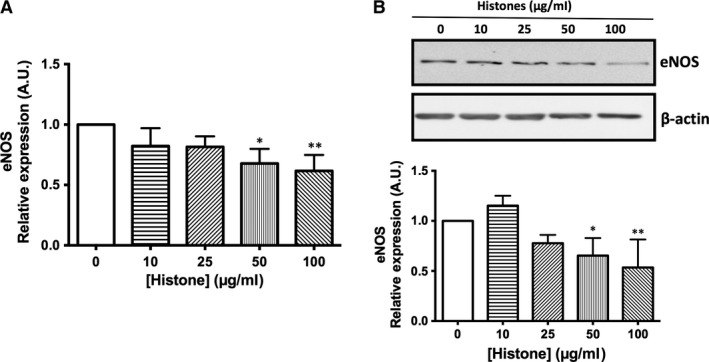
Extracellular histone‐treated HUVECs show decreased eNOS expression. (**A**) HUVEC exposed to increasing concentrations of histones (10–100 μg/ml) for 4 hrs. Relative eNOS expression was determined by qRT‐PCR. Data are expressed as mean ± S.E.M. of *n* = 5–7 from three to five independent experiments. (**B**) Protein extracts (20 μg protein) from cultured HUVEC incubated at different concentrations of histones for 4 hrs were loaded on SDS‐PAGE gels and analysed by Western blotting using anti‐eNOS. β‐actin was used as loading control. One representative experiment of three performed is shown. Relative levels assessed by densitometry are presented. *P < 0.05 and **P < 0.01 *versus* 0 μg/ml.

As described above, extracellular histones decreased NO production in HUVEC (Fig. 1C). To evaluate the synthetic pathway of NO, eNOS mRNA and protein expression were determined in HUVEC exposed to increasing concentrations of histone during 4 hrs. eNOS mRNA levels shown a dose‐dependent decrease at 50 and 100 μg/ml of histones, 32 ± 5% (*P* < 0.01) and 38 ± 5% (*P* < 0.05), respectively (Fig. 3A), result supported by eNOS protein expression (Fig. 3B). Relative levels assessed by densitometry reveal a significant decrease in eNOS protein expression at 50 and 100 μg/ml histones (*P* < 0.05).

### Role of COX‐2 activity in NO production in histone‐treated HUVEC

**Figure 4:**
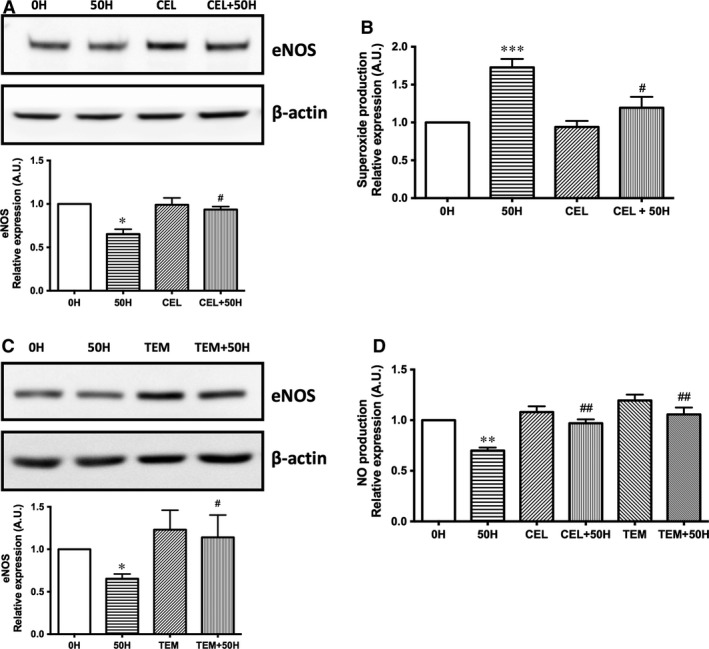
COX‐2, through anion superoxide production, is involved in NO production decrease in extracellular histone‐treated HUVEC. (**A**) HUVECs were exposed to 50 μg/ml of histones (50H) for 4 hrs after 1 hr incubation with celecoxib (CEL), and protein extracts (20 μg protein) were loaded on SDS‐PAGE gels and analysed by Western blotting using anti‐eNOS. β‐actin was used as loading control. One representative experiment of five performed is shown. Relative levels assessed by densitometry are presented. (**B**) Intracellular superoxide levels were determined by DHE oxidation as described in Materials and Methods. Results (mean ± S.E.M. of *n* = 4–5 from three to five independent experiments) (**C**) Histone (50 μg/ml)‐treated HUVECs (50H) were incubated with tempol (TEM), and protein extracts (20 μg protein) were loaded on SDS‐PAGE gels and analysed by Western blotting using anti‐eNOS. β‐actin was used as loading control. One representative experiment of five performed is shown. Relative levels assessed by densitometry are presented. (**D**) HUVEC incubated with celecoxib (CEL) and tempol (TEM) and treated with 50 μg/ml of histones (50H) for 4 hrs were preloaded for 40 min. with the NO probe DAF‐FM to NO production determination. Data are expressed as mean ± S.E.M. of *n* = 5–6 from three to four independent experiments. *P < 0.05; **P < 0.01; ***P < 0.001 *versus* histones 0 μg/ml and ^*#*^ *P* < 0.05; ^*##*^ *P* < 0.01 *versus* histones 50 μg/ml.

As stated before, histones affect COX and eNOS pathways. Previous reports described an interaction between both pathways 17. To investigate whether COX activity is related to NO pathway down‐regulation, histone‐treated HUVECs were incubated with the COX‐2 selective inhibitor celecoxib (10 μmol/l) and eNOS expression was determined. Results indicated that COX‐2 inhibition significantly reversed the reduction in eNOS protein levels induced by 50 μg/ml histones (*P* < 0.05, Fig. 4A).

Moreover, COX have also been described as superoxide (O_2_^−^)‐generating enzymes 18. Thus, we investigated whether COX‐2 activity induced O_2_^−^ production in histone‐treated HUVEC. Extracellular histones (50 μg/ml) induced O_2_^−^ production (73 ± 11%, *P* < 0.001) that was reversed by the inhibitor of COX‐2, celecoxib (10 μmol/l, Fig. 4B), suggesting an involvement of COX‐2 in the O_2_^−^ production induced by histones. To further check whether O_2_^−^ was affecting eNOS expression, incubation with the superoxide dismutase mimetic agent tempol (100 μmol/l) reversed eNOS protein levels in 50 μg/ml histone‐treated endothelial cells (*P* < 0.05, Fig. 4C).

Finally, the involvement of COX‐2‐dependent O_2_^−^ production in NO release by histone‐treated HUVEC was evaluated. The effect of COX‐2 inhibition counteracted the reduced levels of NO production induced by 50 μg/ml histones (*P* < 0.05, Fig. 4D). Similar effect was observed after treatment with tempol (100 μmol/l), which also abrogated NO decreased levels induced by 50 μg/ml extracellular histones (*P* < 0.05, Fig. 4D). These results reinforced the role of increased O_2_^−^ produced by COX‐2 in decreased NO levels.

## Discussion

In the present study, we demonstrated that extracellular histones are implicated in the release of vasoactive mediators in human vascular endothelial cells. First, histone‐treated HUVEC showed an increment in PGI2 production in a dose‐dependent manner. For TXA2 production, the decrease was only significant at 100 μg/ml. Second, extracellular histones increased COX‐2 and PGIS mRNA and protein expression, decreased COX‐1 mRNA levels but not protein expression and did not change TXAS expression. These results suggest an up‐regulation in COX‐2‐PGIS‐PGI2 pathway in those HUVEC exposed to extracellular histones. Third, extracellular histones decreased eNOS expression and NO production in HUVEC. Fourth, we identified COX‐2 as an O_2_^−^‐generating enzyme when HUVECs were exposed to extracellular histones and provide new insights on the mechanism by which COX‐2 activity may interact with NO production through O_2_^−^‐ generation. Altogether, our results suggest a key role of extracellular histones in the modulation of endothelial‐dependent factors, such as prostanoid and NO production, and may explain endothelial cell dysfunction observed in histone‐mediated pathologies.

Extracellular histones have been implicated in organ injury after trauma 2, autoimmune diseases 19, ischaemic heart disease 20 or sepsis 5. As a consequence of the endothelium location, the response trigged by endothelial cells to the circulating histone binding is a crucial event in the development of the histone‐induced injuries. In this regard, it has been recently described that injuries at lungs and liver induced after extracellular histones challenge are primarily mediated through endothelial damage 21 and induced barrier dysfunction 22. Increased calcium influx 23 and up‐regulation of adhesion molecules 24 have also been observed in histone‐treated endothelial cells.

Extracellular histone concentration has been measured in different experimental models. For example, 200 μg/ml of circulating histones was detected in an acute lung injury mice model 25 or 15 μg/ml of H3 in an E.coli‐induced sepsis model in baboons 5. Moreover, similar range of circulating histones concentrations has been reported in patients with blunt traumatic lung injury after 4 hrs (10 to 230 μg/ml) 2 and has been detected in plasma of human patients with sepsis (70 μg/ml) 5. Accordingly, we used a concentration range of extracellular histones (10–100 μg/ml) that showed dose‐dependent changes on the endothelial response studied from 50 μg/ml. Nevertheless, we should note that there exists a discordance in the values of circulating histones levels and those use in experimental models, as other authors have reported levels ranging from 0.01 ng/ml to 1600 ng/ml for sepsis and severe sepsis 26, 27. The wide range of circulating histones in patients suffering different inflammatory diseases described in previous works strongly suggest the need to determine the exact amount of toxic histones. In addition, as stated by Semeraro *et al*. 28, plasma concentrations may underestimate the local amount of histones found at specific sites of cellular release, where they could be much higher.

Our results demonstrate an up‐regulation of the COX‐2‐PGIS pathway and increased synthesis of PGI2 in response to increasing concentrations of extracellular histones. Prostanoids are essential endothelial mediators for maintaining the vascular homeostasis 29. Among them, PGI2 and TXA2 mediate opposite roles in vascular tone and platelet aggregation. Importantly, PGI2 is the main prostanoid synthesized by vascular endothelium, playing a crucial role as regulator of correct vascular function 30. However, PGI2 overregulation can produce vasodilation and shock 31. PGIS is constitutively expressed in endothelial cells where it couples with COX‐1, although COX‐2‐dependent PGI2 production by endothelial cells has been reported to be modulated *in vitro* by inflammatory cytokines 10. PGI2 has also been considered as an endothelial mediator having cytoprotective properties 32. Different studies have reported that PGI2 action serves to protect endothelial cells from apoptosis both *in vitro* and *in vivo* conditions 33, 34. As extracellular histones triggered endothelial cell death using the same concentration range used in our study 23, the increment in PGI2 release observed could be a compensatory action.

Regarding NO pathway, extracellular histones significantly decrease at 50 and 100 μg/ml the production of NO by HUVEC. We found that NO reduction in histone‐treated HUVEC is due, at least in part, to the decrease in eNOS gene and protein expression. In addition to its vasodilatory effect, NO has anti‐inflammatory function and protects against vascular injury and leucocyte adhesion to the endothelium 35, 36. In this regard, decreased NO production levels observed after extracellular histones exposition were in agreement with histone‐dependent actions observed in endothelial cells, such as an increased cell adhesion molecules expression on the cell membrane 24 and neutrophil recruitment 37.

Since Salvemini *et al*. 38 described an interaction of NO and COX enzymes in 1993, several studies support a potential ‘crosstalk’ between both prostanoid and NO pathways 39, 40. COX activity regulation by NO has been described with conflicting results 41, 42 either as positive or as negative regulation of COX activity, and it seems to depend on basal levels of NO released and the cell type used in each study 43. On the other hand, prostanoid biosynthesis has been also found to modulate NO production, as ibuprofen, a well‐known COX inhibitor, increased NO production in arterial endothelial cells 44. In addition, endothelial cell dysfunction or other NO‐depleting situations have been related to a compensatory PGI2 production 45. The interaction between both pathways has also been related to cellular redox status as reactive oxygen species, such as O_2_^−^, can regulate NO bioavailability 46.

O_2_^−^ are released during COX activity as a consequence of their ability to co‐oxidize substances such as NADPH 18. In this regard, COX‐2 has been demonstrated to be an important source of vascular O_2_^−^ production under inflammatory conditions 47, 48, 49. In our study, histone‐treated HUVEC showed an increment in COX‐2 expression, along with an enzymatic activity, in accordance with the increased PGI2 levels observed. Moreover, using celecoxib as a specific inhibitor of COX‐2, we demonstrate that COX‐2 is involved in O_2_^−^ production increment induced when HUVECs are exposed to extracellular histones, consistent with earlier studies using superoxide dismutase in hepatic endothelial cells under inflammatory conditions 50.

Our findings demonstrate that incubation of histone‐treated endothelial cells with celecoxib restored eNOS expression levels. These results are in accordance with those obtained by Fleenor *et al*. 51 where O_2_^−^ depletion restored eNOS expression. In addition, histone‐treated endothelial cells exposed to tempol, a superoxide dismutase mimetic, also restored eNOS expression levels, suggesting a role of COX‐2‐dependent O_2_^−^ in the results observed. We have also shown that inhibition of COX‐2 with celecoxib and decreases of O2‐ by tempol restore NO levels induced by extracellular histones. Similar results were obtained in indomethacin‐treated ageing aortas where NO bioavailability was restored after COX inhibition 17.

**Figure 5:**
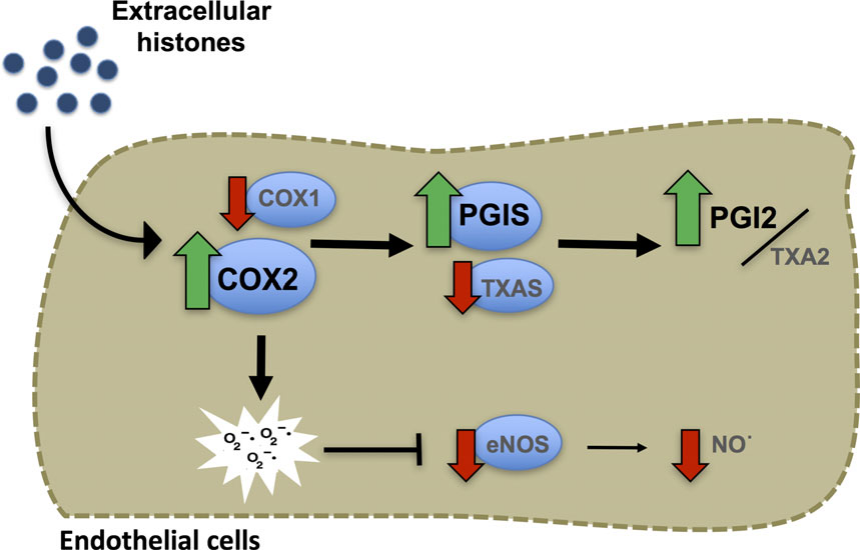
Extracellular histones modulate prostanoid and NO pathways in human endothelial cells. Histone‐treated endothelial cells show higher PGI2/TXA2 ratio through an increment of PGI2 production *via* up‐regulation of COX‐2‐PGIS axis. Extracellular histones increase superoxide levels, due to COX‐2 activity, and contribute to a decreased NO bioavailability.

In conclusion, our findings provided evidence that extracellular histones induce concentration‐dependent changes in the two main vasoactive mediators, resulting in a decrease in NO levels and a shift in prostanoid release (Fig. 5). Histone‐treated endothelial cells show higher PGI2/TXA2 ratio through an increment of PGI2 production *via* up‐regulation of COX‐2‐PGIS pathway. Moreover, the increase in intracellular superoxide levels observed in histone‐treated HUVEC, at least in part produced by COX‐2 activity, contributes to a decreased NO bioavailability. Therefore, the molecular mechanisms described in this work could provide new insight on vascular modulation in pathologies in which extracellular histones are involved.

## Conflict of interest statement

The authors confirm that there are no conflict of interests.
